# Supplementary material for: Identification of Genetic Markers and Genes Putatively Involved in Determining Olive Fruit Weight
Source: Plants (Basel). 2022 Dec 29;12(1):155. doi: 10.3390/plants12010155 (PMC9823435; doi:10.3390/plants12010155)
Supplement: Supplementary file 1 [file plants-12-00155-s001.zip › plants-2059441-supplementary.pdf]

Table S1: Mean olive fruit fresh weight of varieties and wild trees used in the GWAS analysis.

Variety	Fresh fruit weight (g)
Abbadi Abou Gabra-842	6,65
Abou Kanani	6,61
Abou Satl Mohazam	7,17
Arbequina	1,41
Barnea	3,25
Barri	2,19
Chemlal de Kabilye	1,52
Dokkar	0,70
Fishomi	4,28
Forastera de Tortosa	2,62
Frantoio	2,33
Grappolo	2,02
Hojiblanca	4,31
Jabali	4,39
Kalamon	4,01
Klon-14-1812	2,00
Koroneiki	0,61
Leccino	2,98
Lechin de Sevilla	3,04
Lianolia Kerkyras	1,43
Maarri	2,48
Majhol-1013	3,91
Majhol-152	1,69
Manzanilla de Sevilla	4,00
Manzanillera de Huercal Overa	2,83
Mari	3,08
Mastoidis	2,06
Mavreya	1,34
Menya	1,53
Morrut	3,55
Myrtolia	1,54
Ocal	5,47
Picual	3,97
Picudo	4,76
Piñonera	1,26
Royal de cazorla	3,51
Temprano	5,64
Uslu	2,53
Verdial de Velez-Malaga-1	2,79
Wild 1	0,97
Wild 11	0,63
Wild 2	0,65
Wild 3	0,63
Wild 4	0,69
Wild 5	0,89
Wild 6	0,41
Wild 7	0,73
Wild 8	1,55
Wild 9	0,88
Zarza	2,42

(G/A)	(A/A)
1,41462963	0,801039216
0,547619048	1,035424242
1,627857143	0,896862745
2,04	0,873148148
0,805345455	1,495763889
1,126607143	0,683263889
1,486428571	2,031428571
0,927555556	1,68452381
0,988095238	1,837222222
1,774325397	1,068205128
1,672460317	0,969102564
1,108333333	1,69505291
1,647238095	1,564375
0,811107955	0,428510101
1,08352381	
1,604107143	
1,550416667	
1,638095238	
1,506166667	
1,008002137	
1,765416667	

(G/A)	(A/A)	t-student
1,33968244	1,2188516	**0,42776911**
0,39796477	0,48913676	

Kolmogorov-Smirnov	
	Normal
DMAS	0,122679
DMENOS	0,118372
DN	0,122679
Valor-P	0,667998

4 GMs equal
pos. 881555
pos. 881593
pos. 881615
pos. 881750

GM0029B

(C/C)	(C/G)	
0,909555556	1,41462963	
0,801039216	0,547619048	
1,035424242	0,896862745	
0,873148148	2,04	
1,627857143	1,495763889	
0,805345455	1,126607143	
0,97	1,486428571	
1,392857143	0,927555556	
1,567916667	0,988095238	
0,88089899	0,683263889	
1,647238095	1,774325397	
1,068205128	2,031428571	
1,604107143	1,672460317	
1,638095238	1,68452381	
1,69505291	1,108333333	
0,428510101	1,837222222	2 GMs equal
1,765416667	0,811107955	pos. 890759
	1,08352381	pos. 890780
	1,550416667	
	0,969102564	
	1,506166667	
	1,564375	
	1,008002137	

(C/C)	(C/G)	t-student
1,21827458	1,31338322	**0,48646997**
0,41611713	0,42812308	

Kolmogorov-Smirnov	
	Normal
DMAS	0,18357
DMENOS	0,135069
DN	0,18357
Valor-P	0,134994

GM0029C

(G/A)	(G/G)
0,909555556	0,801039216
1,41462963	0,547619048
1,035424242	0,896862745
1,627857143	0,873148148
2,04	0,805345455
1,495763889	1,126607143
1,567916667	1,486428571
1,68452381	0,927555556
1,108333333	0,988095238
1,647238095	0,683263889
0,811107955	1,774325397
1,08352381	0,97
1,550416667	1,392857143
1,69505291	0,88089899
1,765416667	2,031428571
	1,672460317
	1,837222222
	1,068205128
	1,604107143
	0,969102564
	1,638095238
	1,506166667
	1,564375
	1,008002137
	0,428510101

(G/A)	(G/G)	t-student
1,42911736	1,17926887	0,06808259
0,35628492	0,4343995	

Kolmogorov-Smirnov	
	Normal
DMAS	0,133048
DMENOS	0,0961888
DN	0,133048
Valor-P	0,488766

4 GMs equal

pos. 468399
pos. 468479
pos. 468627
pos. 491341

GM0091

(C/C)	(C/T)
0,80103922	0,909555556
0,54761905	1,41462963
1,03542424	0,896862745
0,92755556	0,873148148
0,98809524	1,627857143
0,68326389	2,04
0,97	0,805345455
1,39285714	1,495763889
1,56791667	1,126607143
0,88089899	1,486428571
1,10833333	1,774325397
1,83722222	2,031428571
1,06820513	1,672460317
1,08352381	1,68452381
1,60410714	1,647238095
1,55041667	0,811107955
1,50616667	0,959230769
1,69505291	1,638095238
	1,564375
	1,008002137
	1,765416667

(C/C)	(C/T)	t-student
1,18042766	1,39201915	**0,10265119**
0,37484817	0,40888614	

2 GMs equal

pos. 495009

pos. 497676

Kolmogorov-Smirnov	
	Normal
DMAS	0,116928
DMENOS	0,109455
DN	0,116928
Valor-P	0,660526

GM0148

(A/A)	(A/G)
1,41462963	0,909555556
0,547619048	0,801039216
1,035424242	0,896862745
0,873148148	1,126607143
1,627857143	1,486428571
2,04	0,927555556
0,805345455	0,988095238
1,495763889	0,97
0,683263889	1,392857143
1,774325397	1,567916667
0,88089899	2,031428571
1,68452381	1,672460317
1,647238095	1,108333333
1,837222222	1,604107143
1,068205128	1,550416667
0,811107955	0,969102564
1,08352381	1,638095238
1,69505291	1,506166667
1,008002137	1,564375
1,765416667	

(A/A)	(A/G)	t-student
1,28892843	1,30060018	**0,92932413**
0,45321355	0,35401538	

2 GMs equal
pos. 1194745
pos. 1194771

Kolmogorov-Smirnov	
	Normal
DMAS	0,17956
DMENOS	0,154538
DN	0,17956
Valor-P	0,161793

GM0193

(C/T)	(C/C)
1,41462963	0,909555556
1,035424242	0,801039216
0,873148148	0,547619048
1,627857143	0,896862745
1,495763889	2,04
1,486428571	0,805345455
0,927555556	1,126607143
0,683263889	0,988095238
1,774325397	0,97
1,567916667	1,392857143
2,031428571	0,88089899
1,68452381	1,672460317
1,647238095	1,108333333
1,837222222	1,068205128
0,811107955	1,604107143
1,08352381	0,969102564
1,550416667	1,506166667
1,638095238	1,564375
1,69505291	1,008002137
	0,428510101
	1,765416667

(C/T)	(C/C)	t-student
1,41394328	1,1454076	0,04200857
0,38916331	0,41508229	

3 GMs equal
pos. 643067
pos. 643091
pos. 643095

Kolmogorov-Smirnov	
	Normal
DMAS	0,0747663
DMENOS	0,0878031
DN	0,0878031
Valor-P	0,917379

GM0306

(A/A)	(A/G)	(G/G)
0,909555556	0,896862745	0,547619048
1,41462963	1,627857143	1,035424242
0,801039216	2,04	0,873148148
1,774325397	0,805345455	1,495763889
1,108333333	1,126607143	1,486428571
1,08352381	0,927555556	0,683263889
1,638095238	0,988095238	1,392857143
	0,97	0,811107955
	1,567916667	0,969102564
	0,88089899	1,008002137
	2,031428571	0,428510101
	1,672460317	
	1,68452381	
	1,647238095	
	1,837222222	
	1,068205128	
	1,604107143	
	1,550416667	
	1,506166667	
	1,69505291	
	1,564375	
	1,765416667	

(A/A)	(A/G)	(G/G)
1,24707174	1,42989782	0,97556615
0,36919505	1,45528045	0,36273687

t-student

AA/AG	AG/GG	AA/GG
**0,28664562**	0,00308343	**0,14364555**

t-student

(AA o AG)/GG

0,0026159

1 GM

pos. 871356

Kolmogorov-Smirnov

	Normal
DMAS	0,155334
DMENOS	0,0739334
DN	0,155334
Valor-P	0,290655

GM2874

(G/A)	(G/G)	(A/A)
1,035424242	0,909555556	0,801039216
1,126607143	1,41462963	0,547619048
1,486428571	1,627857143	0,896862745
0,683263889	2,04	0,873148148
1,647238095	0,988095238	0,805345455
1,837222222	1,774325397	1,495763889
1,638095238	0,97	0,927555556
1,69505291	2,031428571	1,392857143
1,564375	1,672460317	1,567916667
	1,68452381	0,88089899
	1,068205128	1,108333333
	1,08352381	0,811107955
	1,604107143	1,765416667
	1,550416667	
	0,969102564	
	1,506166667	
	1,008002137	
	0,428510101	

Kolmogorov-Smirnov

	Normal
DMAS	0,100487
DMENOS	0,0756661
DN	0,100487
Valor-P	0,814036

(G/G)	(G/A)	(A/A)
1,35171722	1,41263415	1,06722037
1,37772672	0,37921922	0,36804343

	t-student	
GA/GG	GG/AA	GA/AA
0,7259066	0,0672949	0,04503586

t-student
(GG o AG)/AA
0,39732118

1 GM
pos. 640676

GM3346

(A/A)	(A/G)	(G/G)
1,41462963	0,896862745	0,909555556
1,035424242	1,627857143	0,801039216
0,873148148	0,805345455	0,547619048
2,04	1,486428571	1,495763889
0,927555556	0,683263889	1,126607143
0,988095238	1,774325397	0,969102564
2,031428571	0,97	1,564375
1,672460317	1,392857143	0,428510101
1,68452381	1,567916667	
1,647238095	0,88089899	
1,068205128	1,108333333	
1,604107143	1,837222222	
1,765416667	0,811107955	
	1,08352381	
	1,550416667	
	1,638095238	
	1,506166667	
	1,69505291	
	1,008002137	

(A/A)	(A/G)	(G/G)
1,442479427	1,280193523	0,980321564
0,417333519	1,301489677	0,406588927

t-student

AA/AG	AG/GG	AA/GG
0,262393428	0,208655148	0,022305378

t-student

(AA o AG)/GG
0,058104662

Kolmogorov-Smirnov

	Normal
DMAS	0,0762693
DMENOS	0,0810266
DN	0,0810266
Valor-P	0,955414

1 GM
pos. 582095

GM3361.1

(A/A)	(A/G)
1,41462963	0,909555556
1,035424242	0,801039216
0,873148148	0,547619048
1,627857143	0,896862745
2,04	1,495763889
0,805345455	1,126607143
1,486428571	0,683263889
0,927555556	0,97
0,988095238	1,392857143
1,774325397	1,567916667
2,031428571	0,88089899
1,672460317	1,837222222
1,68452381	0,811107955
1,108333333	0,969102564
1,647238095	1,638095238
1,068205128	1,69505291
1,08352381	1,564375
1,604107143	1,008002137
1,550416667	
1,506166667	
1,765416667	

(A/A)	(A/G)
1,41402998	1,155296795
0,379577234	1,169752162

t-student

0,04382103

Kolmogorov-Smirnov	Normal
DMAS	0,0762693
DMENOS	0,0810266
DN	0,0810266
Valor-P	0,955414

2 GMs equal

pos. 582217

pos. 582274

GM3361.2

(A/A)	(A/C)
1,41462963	0,909555556
1,035424242	0,801039216
0,873148148	0,547619048
2,04	0,896862745
0,927555556	1,627857143
0,988095238	0,805345455
2,031428571	1,495763889
1,672460317	1,126607143
1,68452381	1,486428571
1,647238095	0,683263889
1,068205128	1,774325397
1,604107143	0,97
1,765416667	1,392857143
	1,567916667
	0,88089899
	1,108333333
	1,837222222
	0,811107955
	1,08352381
	1,550416667
	0,969102564
	1,638095238
	1,506166667
	1,69505291
	1,564375
	1,008002137
	0,428510101

(A/A)	(A/C)
1,44247943	1,19134257
0,41733352	1,24250757

t-student
0,05916799

Kolmogorov-Smirnov

	Normal
DMAS	0,0762693
DMENOS	0,0810266
DN	0,0810266
Valor-P	0,955414

1 GM
pos. 582298

GM3361.3

(G/A)	(G/G)
1,41462963	0,909555556
0,801039216	0,547619048
2,04	1,035424242
0,805345455	0,896862745
1,495763889	0,873148148
0,988095238	1,627857143
1,774325397	1,126607143
1,567916667	1,486428571
2,031428571	0,927555556
1,68452381	0,683263889
1,647238095	0,97
1,837222222	1,392857143
0,811107955	0,88089899
1,08352381	1,672460317
0,969102564	1,108333333
1,506166667	1,068205128
1,69505291	1,604107143
1,564375	1,550416667
1,008002137	1,638095238
	0,428510101
	1,765416667

(G/A)	(G/G)
1,40657154	1,15207727
0,41580523	0,39579648

t-student

0,05466632

1 GM

pos. 386986

Kolmogorov-Smirnov

	Normal
DMAS	0,0867479
DMENOS	0,109355
DN	0,109355
Valor-P	0,72513

GM3663

(C/C)	(C/G)
1,41462963	0,909555556
0,547619048	0,801039216
1,035424242	1,126607143
0,896862745	1,486428571
0,873148148	0,927555556
1,627857143	0,988095238
2,04	0,97
0,805345455	1,392857143
1,495763889	1,567916667
0,683263889	2,031428571
1,774325397	1,672460317
0,88089899	1,108333333
1,68452381	1,604107143
1,647238095	1,550416667
1,837222222	0,969102564
1,068205128	1,638095238
0,811107955	1,506166667
1,08352381	1,564375
1,69505291	
1,008002137	
1,765416667	

(C/C)	(C/G)	t-student
1,27025863	1,32303003	**0,68887737**
0,44994687	0,35011127	

1 GM

pos. 422150

Kolmogorov-Smirnov

	Normal
DMAS	0,174048
DMENOS	0,150113
DN	0,174048
Valor-P	0,188386

GM4112

(T/T)	(T/C)
1,41462963	0,909555556
0,801039216	1,035424242
0,547619048	1,495763889
0,896862745	1,126607143
0,873148148	0,927555556
1,627857143	0,988095238
2,04	0,97
0,805345455	1,567916667
1,486428571	0,88089899
0,683263889	2,031428571
1,774325397	1,68452381
1,392857143	1,108333333
1,672460317	0,969102564
1,647238095	1,69505291
1,837222222	0,428510101
1,068205128	1,765416667
0,811107955	
1,08352381	
1,604107143	
1,550416667	
1,638095238	
1,506166667	
1,564375	
1,008002137	

(T/T)	(T/C)
1,3055957	1,22401158
0,42093703	0,42833181

t-student

0,55447228

1 GM

pos. 193259

Prueba de	
	Normal
DMAS	0,16316
DMENOS	0,110843
DN	0,16316
Valor-P	0,237946

GM4403

(C/G)	(G/G)
1,41462963	0,909555556
1,035424242	0,801039216
0,873148148	0,547619048
1,627857143	0,896862745
1,495763889	2,04
1,486428571	0,805345455
0,927555556	1,126607143
1,774325397	0,988095238
1,567916667	0,683263889
2,031428571	0,97
1,68452381	1,392857143
1,647238095	0,88089899
1,837222222	1,672460317
0,811107955	1,108333333
1,08352381	1,068205128
1,550416667	1,604107143
1,638095238	0,969102564
1,69505291	1,506166667
	1,564375
	1,008002137
	0,428510101
	1,765416667

(C/G)	(G/G)
1,45453658	1,12440107
0,35666036	0,41688956

t-student

0,01149312

1 GM

pos. 118649

Kolmogorov-Smirnov	
	Normal
DMAS	0,0949608
DMENOS	0,0984066
DN	0,0984066
Valor-P	0,833339

GM4878.1

(C/C)	(T/C)	T/T
0,909555556	1,41462963	1,486428571
0,801039216	1,035424242	0,927555556
0,547619048	0,873148148	1,774325397
0,896862745	1,627857143	1,567916667
2,04	1,495763889	1,68452381
0,805345455	0,988095238	
1,126607143	0,97	
0,683263889	1,392857143	
0,88089899	2,031428571	
1,672460317	1,647238095	
1,108333333	1,837222222	
1,068205128	0,811107955	
1,604107143	1,08352381	
0,969102564	1,550416667	
1,506166667	1,638095238	
1,564375	1,69505291	
1,008002137		
0,428510101		
1,765416667		

1 GM

pos. 118890

(C/C)	(T/C)	T/T
1,125572163	1,380741306	1,48815
0,372808076	0,443171729	0,332073353

t-student

CC/TT	TC/CC	TC/TT
**0,103841082**	0,077482996	**0,572057872**

t-student

(TT o TC)/CC
0,135311384

Kolmogorov-Smirnov

	Normal
DMAS	0,0949608
DMENOS	0,0984066
DN	0,0984066
Valor-P	0,833339

GM4878.2

(C/T)	(C/C)
1,41462963	0,909555556
0,547619048	0,801039216
0,896862745	1,035424242
1,627857143	0,873148148
0,805345455	2,04
1,486428571	1,495763889
0,988095238	1,126607143
0,97	0,927555556
1,392857143	0,683263889
1,567916667	1,774325397
0,88089899	1,68452381
2,031428571	1,108333333
1,672460317	1,647238095
1,068205128	1,837222222
1,604107143	0,811107955
0,969102564	1,08352381
1,564375	1,550416667
0,428510101	1,638095238
	1,506166667
	1,69505291
	1,008002137
	1,765416667

(C/T)	(C/C)
1,21759441	1,31826284
0,43571927	0,41193176

t-student

0,45830776

1 GM

pos. 23710

Kolmogorov-Smirnov	
	Normal
DMAS	0,186923
DMENOS	0,156989
DN	0,186923
Valor-P	0,122218

GM5420

(T/T)	(T/C)
0,873148148	0,909555556
2,04	1,41462963
0,927555556	0,801039216
0,988095238	0,547619048
2,031428571	1,035424242
1,672460317	0,896862745
1,68452381	1,627857143
1,647238095	0,805345455
1,068205128	1,495763889
1,604107143	1,126607143
1,765416667	1,486428571
	0,683263889
	1,774325397
	0,97
	1,392857143
	1,567916667
	0,88089899
	1,108333333
	1,837222222
	0,811107955
	1,08352381
	1,550416667
	0,969102564
	1,506166667
	1,638095238
	1,69505291
	1,564375
	1,008002137
	0,428510101

(T/T)	(T/C)
1,48201624	1,19366563
0,43661687	0,39287449

t-student

0,05141508

1 GM

pos. 84939

Kolmogorov-Smirnov

	Normal
DMAS	0,148563
DMENOS	0,126968
DN	0,148563
Valor-P	0,343002

GM5641

(A/A)	(A/G)
1,41462963	0,89686275
1,035424242	0,87314815
2,04	0,68326389
1,486428571	0,97
0,927555556	1,39285714
0,988095238	1,56791667
1,774325397	0,88089899
2,031428571	1,83722222
1,672460317	0,81110795
1,68452381	1,63809524
1,108333333	1,69505291
1,647238095	1,00800214
1,068205128	0,90955556
1,08352381	0,80103922
1,604107143	0,54761905
1,550416667	1,62785714
1,506166667	0,80534545
1,564375	1,49576389
1,765416667	1,12660714
	0,96910256
	0,4285101

(A/A)	(A/G)
1,47119231	1,09361086
0,34426459	0,40839954

t-student

0,00323413

1 GM

pos. 73917

Kolmogorov-Smirnov	
	Normal
DMAS	0,0719805
DMENOS	0,103762
DN	0,103762
Valor-P	0,782267

GM6972
